# A bibliometric analysis of published research employing musculoskeletal imaging modalities to evaluate foot osteoarthritis

**DOI:** 10.1186/s13047-022-00549-0

**Published:** 2022-05-20

**Authors:** Prue Molyneux, Sarah Stewart, Catherine Bowen, Richard Ellis, Keith Rome, Matthew Carroll

**Affiliations:** 1 School of Clinical Science, Faculty of Health and Environmental Science, Auckland University of Technology, 90 Akoranga Drive, Northcote, New Zealand; 2 Active Living and Rehabilitation: Aotearoa New Zealand, Health and Rehabilitation Research Institute, School of Clinical Sciences, Auckland University of Technology, Auckland, New Zealand; 3 School of Health Sciences, Faculty of Environmental and Life Sciences, University of Southampton, Southampton, UK; 4 Centre for Sport, Exercise and Osteoarthritis Versus Arthritis, University of Southampton, Southampton, UK

**Keywords:** Imaging modalities, Foot osteoarthritis

## Abstract

**Objectives:**

Temporal and global changes in research utilising imaging to assess foot osteoarthritis is currently unknown. This study aimed to undertake a bibliometric analysis of published research to: (1) identify the imaging modalities that have been used to evaluate foot osteoarthritis; (2) explore the temporal changes and global differences in the use of these imaging modalities; and (3) to evaluate performance related to publication- and citation-based metrics.

**Methods:**

A literature search was conducted using Scopus to identify studies which had used imaging to assess foot osteoarthritis. Extracted data included publication year, imaging modality, citations, affiliations, and author collaboration networks. Temporal trends in the use of each imaging modality were analysed. Performance analysis and science mapping were used to analyse citations and collaboration networks.

**Results:**

158 studies were identified between 1980 and 2021. Plain radiography was the most widely used modality, followed by computed tomography, magnetic resonance imaging (MRI) and ultrasound imaging (USI), respectively. The number of published studies increased over time for each imaging modality (all *P* ≥ 0.018). The most productive country was the United States of America (USA), followed by the United Kingdom and Australia. International authorship collaboration was evident in 57 (36.1%) studies. The average citation rate was 23.4 per study, with an average annual citation rate of 2.1.

**Conclusions:**

Published research employing imaging to assess foot osteoarthritis has increased substantially over the past four decades. Although plain radiography remains the gold standard modality, the emergence of MRI and USI in the past two decades continues to advance knowledge and progress research in this field.

**Supplementary Information:**

The online version contains supplementary material available at 10.1186/s13047-022-00549-0.

## Background

Osteoarthritis is a progressive joint disease involving degradation of articular cartilage, subchondral bone and surrounding soft tissue structures, leading to symptoms of pain and stiffness [1]. Small joints of the feet are often overlooked as a site of involvement relative to other joints commonly affected by osteoarthritis [2, 3]. Population-based epidemiological studies have reported a high prevalence of radiographic osteoarthritis in the feet, with up to 39% of older adults having first metatarsophalangeal joint involvement [2].

Musculoskeletal imaging has an essential role in the diagnosis and assessment of osteoarthritis [4–8]. Plain radiography has traditionally been regarded as the leading modality for the assessment of osseous changes in foot osteoarthritis [9], and there is emerging evidence that assesses the diagnostic sensitivity of radiography and its association to clinical symptoms for foot disorders [10–12]. However, the advent of more advanced techniques, including magnetic resonance imaging (MRI), computed tomography (CT), and ultrasound imaging (USI) have gained increasing recognition for their fundamental role in understanding the pathophysiology of osteoarthritis due to their ability to observe subclinical osseous and soft tissue changes [13–18].

Recommendations published in 2017 by the European League Against Rheumatism (EULAR) highlighted the need for further imaging research into less commonly studied sites of osteoarthritis, such as the foot [19]. However, the global status of collaboration and temporal trends of research employing various imaging modalities to assess osteoarthritis in the foot is currently unknown. Published research is central in providing information and improving knowledge, and publication and citation-based metrics provide a means to monitor the productivity and impact, or influence, of published information [20].

In order to evaluate the scope of research utilising imaging to assess foot osteoarthritis, including global and temporal trends, and performance-based metrics, a bibliometric analysis is required. Bibliometric analyses summarise information to uncover emerging trends in article and journal performance, collaboration patterns, and research constituents [20, 21]. The objectives of this study were to conduct a bibliometric analysis of published literature used to evaluate foot osteoarthritis to: (1) identify the imaging modalities that have been used; (2) explore the temporal changes and global differences in the use of these imaging modalities; and (3) evaluate performance related to publication- and citation-based metrics.

## Methods

The procedure and best practice guidelines proposed by Donthu et al. [20] were used to frame the bibliometric analysis methodology. The techniques for bibliometric analysis manifest across two categories: (1) performance analysis and (2) science mapping [20]. In essence, performance analysis examines the contributions of research constitutes, which is descriptive in nature. Whereas science mapping focuses on the relationships between research constitutes [20, 22–24]. Both techniques were used to address the study objectives. Performance analysis was used to examine publication-related metrics and recognises the influence of various constituents (authors, institutions/affiliations, countries, and journals) on publication performance which is measured using a range of metrics (total publications, number of contributing authors, co-authored publications, number of active years of publication, total citations, average citations and annual citation rate). Science mapping was used to examine the relationships between research constituents [22–24]. Two science mapping techniques were used; (1) citation analysis, to identify the most influential publications in the field and (2) co-authorship analysis, to examine the social interactions or relations among authors and their affiliations and equivalent impacts on the development of the research field [20].

### Search strategy

The search strategy required for a bibliometric analysis is similar to that of a systematic review. However, it must identify a large enough volume of articles to warrant bibliometric analysis and fulfil the requirements of the analysis techniques, yet also be focused enough to remain in the dedicated research field [20]. An electronic search was designed and conducted by two authors (PM and SS) in August 2021 using the search terms displayed in Supplementary File 1. The two most commonly utilised databases being Scopus®, and Web of Science®. It is accepted practice when using metadata for bibliometric analysis to use either Scopus® or Web of Science® [20]. It is recommended to select one appropriate database to mitigate the need for consolidation of data, minimising unnecessary action items can help to mitigate potential human errors [20]. The Scopus® database (Elsevier, Amsterdam, Netherlands) was selected as it has the largest abstract and citation database of research literature [25]. As of January 2020, Scopus® had in excess of 25,100 active titles and over 550 articles in press [26]. Additionally Scopus® includes a more expanded spectrum of journals than PubMed and Web of Science® [27]. There were no restrictions on date, allowing a search for studies published from the database’s earliest record (1963) through to August 2021. The electronic search was supplemented with hand-searching of references lists from included studies and review articles to identify additional eligible papers.

### Study selection

The titles and abstracts of all identified studies were downloaded from the Scopus® database and exported into Rayyan (http://rayyan.qcri.org), an online literature review application [28], suitable for bibliometric analysis. In the first stage of selection the titles and abstracts were independently screened by two authors (PM and SS). Studies were included if they used imaging to assess the foot in patients with foot osteoarthritis (inclusive of post-traumatic foot osteoarthritis), for the purpose of recruitment or as a research outcome, reported original research findings, and were published in English. Studies were excluded if they did not report original research findings, including reviews, case reports, commentaries, letters, non-human studies, conference proceedings, or editorials, or did not report which imaging modality was used to assess the foot. Studies that evaluated ankle osteoarthritis but did not also include the assessment of foot osteoarthritis were excluded. To determine the final articles for inclusion, the full texts of all articles included at the title/abstract screening stage were retrieved and reassessed against the criteria. Conflicts were discussed between the two authors (PM and SS) until consensus was achieved.

### Data extraction

All studies were imported into Biblioshiny (based on R version 3.6.1, Bibliometrix package version 2.2.1; University of Naples Federico II, Naples, Italy, 2016) [29] for data extraction. The following bibliometric indicators were extracted from each study: year of publication, journal name, journal impact factor (IF) (in prior year, 2020 using the Web of Science Journal Citation Reports™ tool (Clarivate Analytics, Philadelphia, Pennsylvania, USA), number of citations (determined the Scopus® database (Elsevier)), author names, total authors per manuscript, and institutional affiliation of each author. The extent of collaboration for each study was also determined based on four categories: (1) “international collaboration” in which studies involved collaboration with international authors; (2) “bi-national collaboration” in which studies originated from authors affiliated to only two institutions/affiliations from the same country; (3) “multi-national collaboration” in which studies were authored by researchers from three or more institutions/affiliations from the same country; and (4) “no collaboration” in which all authors were affiliated with the same institution [30, 31].

Additional data from each included study were also extracted into a standardised Microsoft Excel spreadsheet (Version 2016, Microsoft Corp., Redmond, Washington, USA), including: the imaging modality/modalities used (plain radiography, CT, MRI or USI), the specific joints in the foot that were assessed, the reason for assessment (outcome measure vs. recruitment screening tool) and the study design. Finally, study design was classified using the Oxford Centre for Evidence-Based Medicine (OCEBM) Levels of Evidence [32], in which randomised controlled trials are classified as Level 2, cohort/longitudinal studies as Level 3, and case-series and case-control studies as Level 4 (note: systematic reviews (Level 1) and studies involving mechanism-based reasoning (Level 5) were not eligible for inclusion in the current analysis).

### Data analysis

Descriptive statistics were used to summarise the study characteristics and data related to each imaging modality. Bibliometric data were analysed separately for each imaging modality using the bibliometrix package, Biblioshiny. Studies utilising more than one imaging modality to assess osteoarthritis in the foot were included in analyses for each imaging modality used. As described above, the bibliometric analysis included both a performance analysis (to determine the total publications, number of contributing authors, co-authored publications, number of active years of publication (number of years from publication to 2021), total citations, average citations and annual citation rate); and science mapping (to undertake a citation analysis and co-authorship analysis). The citation analysis included evaluating the difference in publication performance of each imaging modality over time. In this analysis, the impact of a publication is determined by the number of citations that it receives. The analysis enables the most influential publications in a research field to be ascertained [20]. The co-authorship analysis also included generation of a collaboration world map representing the extent of international author collaborations across the included studies.

In addition, to complement the bibliometric analysis and further explore publication trends over time, linear regression models were used to analyse temporal trends in the use of each imaging modality over time using SPSS (for example, increased or decreased imaging modality use over time) (Version 26.0. IBM Corp, Armonk, NY). Studies were grouped into decades, based on year of publication. The 2021 publication year was excluded from this analysis due to incomplete data (i.e., data was collected up to and including August 2021).

## Results

### Characteristics of included studies

A total of 1905 studies were initially identified, of which 158 studies satisfied the inclusion criteria and were included in the final analysis (Supplementary File 2). Characteristics of the included studies are displayed in Table 1. The included studies were published between 1980 and 2021 with an annual percentage growth rate of 9.6%. The majority of included studies employed plain radiography to assess the foot, followed by CT, MRI and USI. Twenty-four studies used two imaging modalities, and the remaining 134 studies used a single imaging modality. When characterised by the Oxford 2011 Level of Evidence, eight (5.1%) studies were Level 2, 58 (36.7%) were Level 3, and 92 (58.2%) were Level 4. One hundred and thirteen (71.5%) studies used imaging to assess a study outcome, nine (5.7%) studies used imaging in the recruitment process to screen participants for inclusion, and 36 (22.8%) used imaging as both a screening tool and outcome measure. The most common foot joint scanned was the first metatarsophalangeal joint (*n* = 64 studies, 40.5%). Supplementary File 3 presents the proportion of studies assessing each specific joint across each imaging modality.

**Table 1:** Characteristics of the included studies (*n* = 158)

Timespan, year		1980 to 2021
Annual percentage growth rate, %		9.6%
Oxford 2011 Levels of Evidence, n (%)	Level 2	8 (5.1%)
	Level 3	58 (36.7%)
	Level 4	92 (58.2%)
Imaging modality used, n (%)^a^	Plain radiography	140 (88.6%)
	CT	24 (15.2%)
	MRI	14 (8.9%)
	USI	4 (2.5%)
Reason for imaging, n (%)	Outcome measure	113 (71.5%)
	Participant recruitment	9 (5.7%)
	Both	36 (22.8%)
Foot joint examined with imaging, n (%)^b^	First metatarsophalangeal joint	64 (40.5%)
	Lesser metatarsophalangeal joints	9 (5.7%)
	Interphalangeal joints	7 (4.4%)
	Subtalar joint	50 (31.6%)
	Midfoot joints	82 (51.9%)
	Not specified	6 (3.8%)

^a^24 studies used more than one imaging modality

^b^39 studies assessed ≥ 2 ft joints

#### Assessment of temporal changes in the use of imaging modalities

**Fig. 1:**
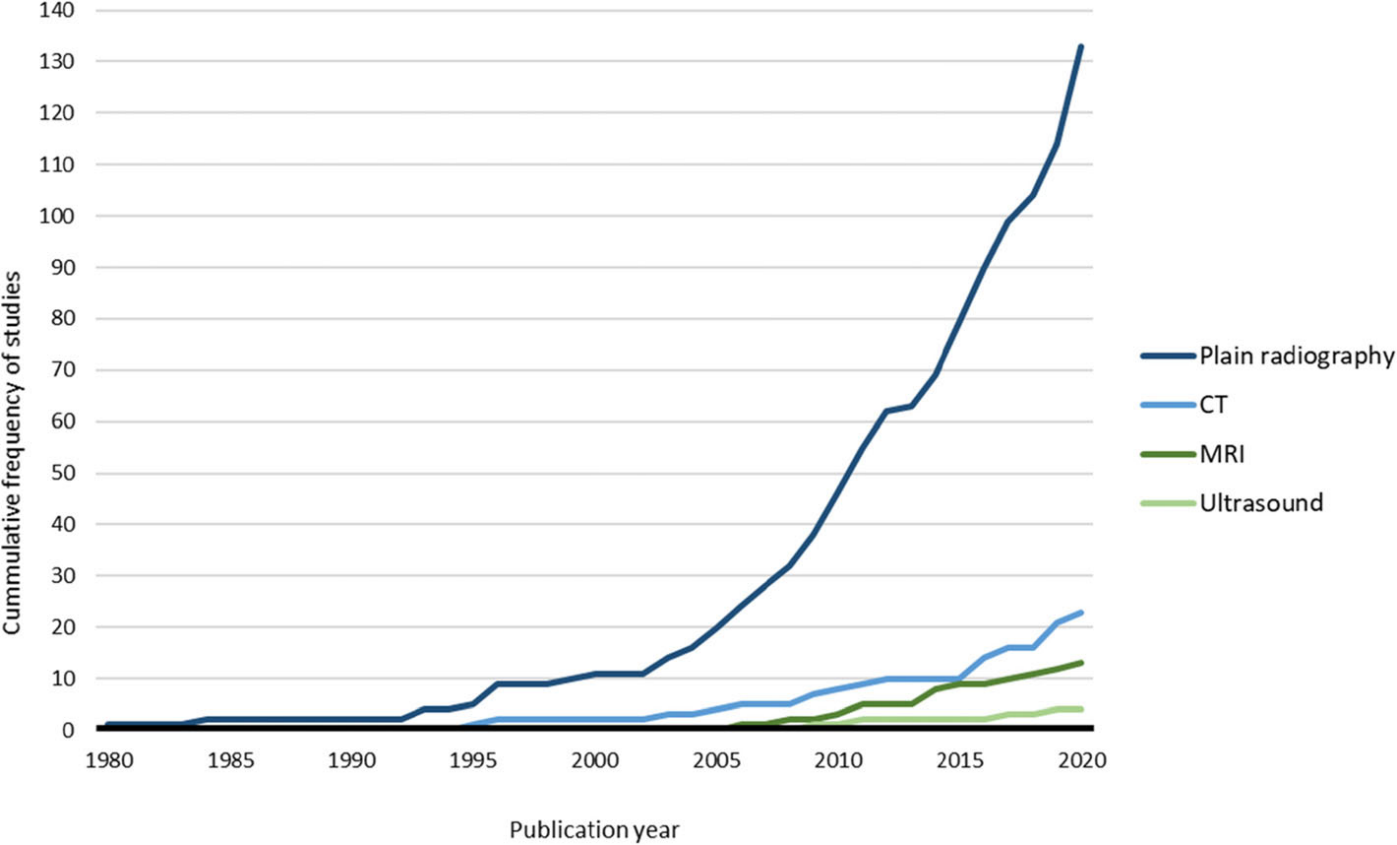
Cumulative frequency of studies published per year for each imaging modality. Note: data from studies using more than one imaging modality (*n* = 24) were counted for each imaging modality used

The earliest study was published in 1980 and the most recent study in 2021. The earliest study published using plain radiography to assess foot osteoarthritis was 1980, while the use of CT did not appear until 1995, MRI until 2006, and USI until 2009 (Fig. 1). This finding is also consistent with the average years from publication to 2021, which was 8.4 years for studies using plain radiography, 8.1 years for CT, 6.9 years for MRI, and 7.0 years for USI. Each modality showed a significant increase in the number of publications across decades for plain radiography (*P* for trend < 0.001), CT (*P* for trend = 0.001), MRI (*P* for trend < 0.001), and USI (*P* for trend = 0.018).

#### Assessment of global differences in the use of imaging modalities

**Fig. 2:**
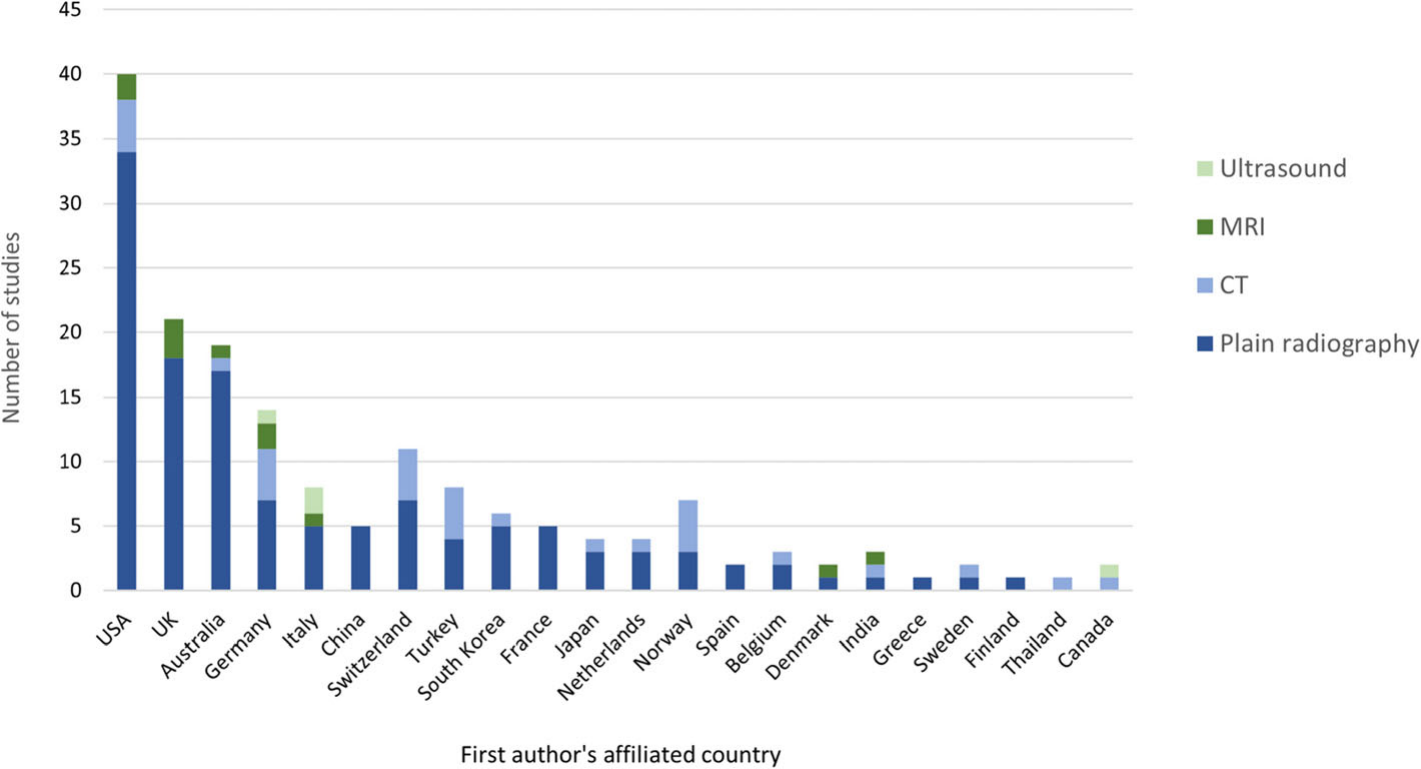
Number of studies associated with the first authors affiliated country for each imaging modality. Note: data from studies using more than one imaging modality (*n* = 24) were counted for each imaging modality used

The 158 included studies were published across 22 different countries based on the first author’s affiliation (Fig. 2). The most productive country was the USA, with 36 (22.8%) included studies based on the first authors affiliation, followed by the United Kingdom, publishing 20 (12.7%), and Australia, publishing 17 (10.8%). The most common first author affiliation for studies using plain radiography was the USA (*n* = 34 studies), while CT studies were associated with first authors from the USA (*n* = 4) and Germany (*n* = 4). The most common first author affiliation for MRI studies was the United Kingdom (*n* = 3 studies), and for USI studies was Italy (*n* = 2 studies) (Fig. 2).

**Fig. 3:**
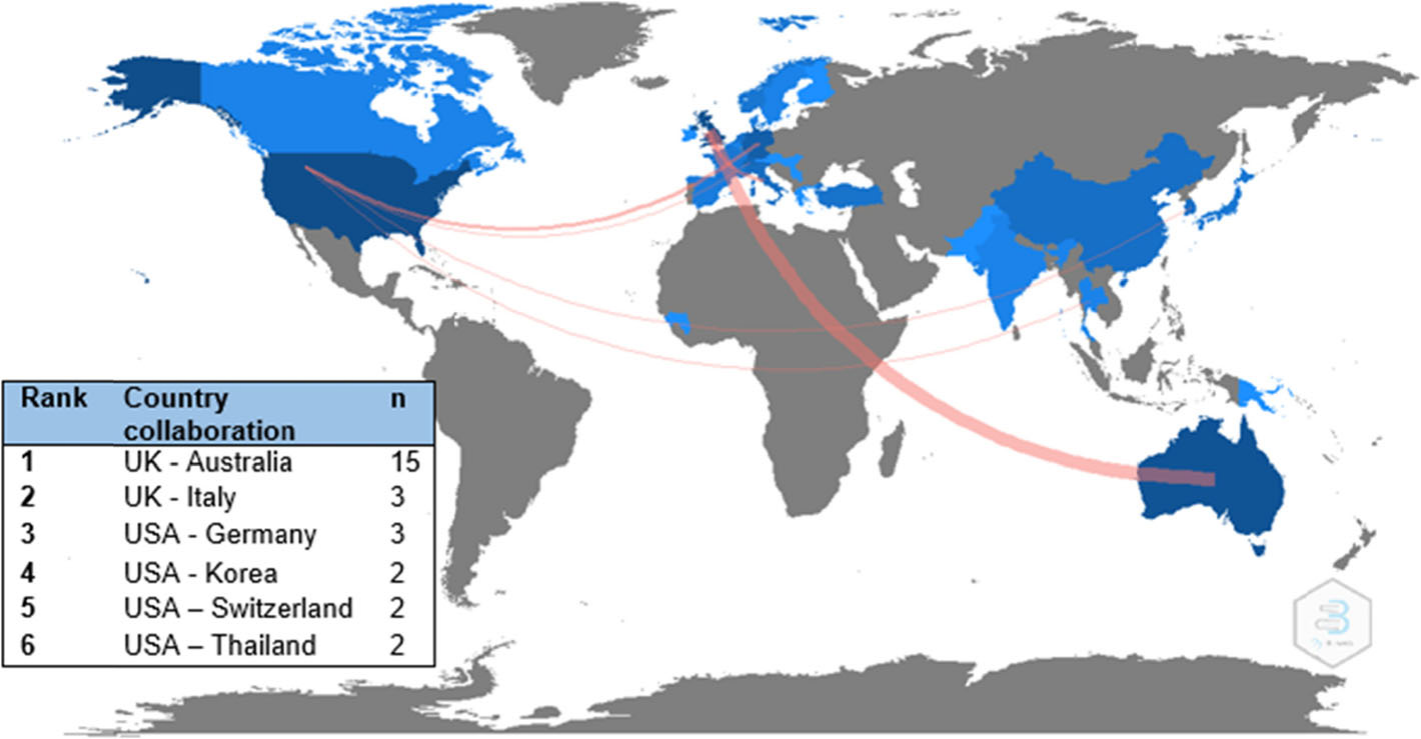
Visual representation of international collaborative authorship networks. Note: Different shades of blue indicate different rates of collaboration between that country with any other country: dark blue = higher collaboration; grey = no collaboration. The red lines indicate countries where there were two or more research collaborations: thicker lines indicate a greater number of collaborations

Fifty-seven (36.1%) of the included articles demonstrated international collaboration, 34 (21.5%) articles had bi-national collaboration, 43 (27.2%) articles had evidence of multi-national collaboration, and 24 (15.2%) articles had no collaborative links outside of a single institution. The most frequent international authorship link occurred between the United Kingdom and Australia (15 articles). A world map displaying international collaborative research links by country is displayed in Fig. 3**.**

#### Performance analysis

##### Citations

The 158 included studies had a total of 3690 citations, with an average citation rate of 23.4 per study (range: 0–202 citations). Fourteen (8.9%) studies received no citations; however, these had recent publication dates in 2021 (*n* = 8 studies) and 2020 (*n* = 6 studies). Total citations, average citations per study, and average annual citation rates per study are displayed in Table 2. Although studies using plain radiography had the highest total citations, studies using CT had the highest average citations per study and highest average annual citation rate. A summary of the top five most cited countries based on the first author’s country for each imaging modality is displayed in Supplementary File 4. The most cited study was published by Paley et al. [33] in 1993 and used plain radiography to assess the hindfoot (*n* = 202 citations). This study represented 5.5% of the total citations for all included studies. The twenty most cited studies represented 50.5% of the total citations. The ten most cited articles are displayed in Supplementary File 5.

**Table 2:** Citations across imaging modalities

		All	Plain radiography	CT	MRI	USI
Number of studies		158^a^	140	24	14	4
Total citations		3690	3337	616	326	56
Average citations per study		23.4	23.8	25.7	23.3	14.0
Average annual citation rate per study		2.1	2.1	2.2	1.3	1.5
Most cited country	Country	United States of America	United States of America	United States of America	United States of America	Canada
	Number of studies	36	34	4	2	1
	Total citations	1724	1538	216	173	29
	Average citations per study	47.6	45.2	54.0	86.5	29.0
	Average annual citation rate per study	3.1	2.9	3.1	6.9	2.2
Most cited author *(first author)*	Author	Menz HB	Menz HB	Stephens HM	El-Rashidy H	Matsos M
	Number of studies	10	10	1	1	1
	Total citations	254	254	104	107	29
	Average citations per study	25.4	25.4	104	107	29
	Average annual citation rate per study	2.8	2.8	4.0	9.7	2.2
Most cited author *(any author)*	Author	Menz HB	Menz HB	Stephens HM	El-Rashidy H	Matsos M
	Number of studies	27	26	1	1	1
	Total citations	560	558	104	107	29
	Average citations per study	20.7	21.5	104	107	29
	Average annual citation rate per study	2.5	2.6	4.0	9.7	2.2
Most cited journal	Journal	Journal of Bone and Joint Surgery-Series A	Journal of Bone and Joint Surgery-Series A	Foot and Ankle International	Foot and Ankle International	Skeletal Radiology
	Number of studies	8	7	9	1	1
	Total citations	931	824	308	107	29
	Average citations per study	116	117	34	107	29
	Average annual citation rate per study	6.0	5.5	2.4	9.7	2.2

^a^24 studies used ≥ 2 imaging modalities

##### Journals

The 158 included studies were published in 64 different journals (Supplementary File 6). *Foot and Ankle International* published the highest proportion of studies (*n* = 24, 15.8%). There were 42 journals (27.6%) where only one of the included articles were published. The journals that published three or more publications accounted for 57.9% of all identified studies. Studies utilising plain radiography were published across 55 different journals, CT across 15 journals, MRI across 14 journals, and USI across four journals. A 2020 IF was available for 51 (79.7%) of the journals where the included articles were published. The mean IF for the included studies was 3.74, median IF was 2.83, with an IF range of 0.51–19.10. The articles published in *Journal of Bone and Joint Surgery-Series A* were the most cited, with a total of 931 citations, average citations per study of 116 and an average annual citation rate per study of 6.0.

##### Authorship and affiliations

A total of 661 authors contributed to the 158 included studies, with a mean of five co-authors per study. Studies using plain radiography had an average author/study count of 5.3, CT had 4.5, and MRI had 5.9. A recent USI study had the largest number of authors listed for a single study (*n* = 24) [30], bringing the average co-authors per study up to 11.8 for studies using USI. Only one study was authored by a single author [34]. Supplementary File 7 displays the ten most published authors based on their appearance as first author, or as an author listed anywhere in the author list. Hylton Menz had the largest number author appearances (including first author and any author), and total citations.

The 158 included studies were conducted across 232 individual affiliations/institutions. Supplementary File 7 shows the top ten most productive affiliations based on the number of published studies associated with the affiliation of any listed author. La Trobe University located in Australia and Keele University in the United Kingdom were the institutions that published the highest numbers of studies (*n* = 27 and *n* = 19, respectively).

## Discussion

This is the first bibliometric analysis of published studies employing musculoskeletal imaging to assess the foot in people with osteoarthritis. The findings have shown a notable increase in the publication of studies in this field over the past four decades. Despite this being a likely generic trend across many healthcare fields, this is of relevance given research in the field of foot osteoarthritis needs to be accelerated [35]. Although plain radiography remains the earliest and most widely used in research to assess foot osteoarthritis, CT, MRI, and USI have become increasingly more common in the past decade.

Research specific to foot osteoarthritis is less advanced than that of other joints, including knee and hip osteoarthritis [36–39]. However, a notable surge in imaging studies for foot osteoarthritis, particularly at the first metatarsophalangeal joint, was noted in the current analysis, the timeline of which is consistent with publication of the 2017 EULAR recommendations to increase research in this field [19]. Although studies using plain radiography have substantially increased over the years, the results from this analysis have shown an increased uptake of more advanced imaging. This is consistent with the reported use of MRI, CT, and USI for general musculoskeletal assessments which have increased 615, 758, and 500%, respectively, over the past two decades [40].

To date, one of the most notable imaging advancements specific to foot osteoarthritis was the development of the La Trobe Radiographic Foot Atlas in 2007 [9]. The impact of this study was evident by its position among the top 10 most cited studies in this analysis. This atlas has led to significant improvements in the ability to consistently estimate prevalence of this condition [41], as well as understand different patterns of foot joint involvement [12]. Although more advanced modalities, including MRI and USI are emerging as more accurate evaluators of both bone and soft-tissue abnormalities in foot osteoarthritis [17, 42], it is likely that plain radiography will continue to remain the gold standard until validity of these more advanced techniques are determined. Ongoing research in this area is crucial in determining the capacity of modern imaging modalities to detect early inflammatory changes that precede osseous involvement, therefore informing more timely management approaches that aim to prevent further structural progression [7, 8, 43, 44].

The USA accounted for the largest volume of published studies, based on the first author’s affiliation, which primarily utilised plain radiography to assess foot osteoarthritis. This finding is comparable with previous bibliometric studies conducted in other medical fields in which the USA is consistently associated the highest research production [45–47]. The USA, despite producing the largest volume of work, had the fewest international collaborations. Studies from this region were published by large numbers of different authors affiliated with several different institutions within the USA. In contrast, studies from the United Kingdom and Australia demonstrated the most frequent international collaborations and tended to involve the same authors affiliated with a small number of institutions. This may reflect the more frequent publication of studies employing CT, MRI and USI in European countries. Clinical-based surveys have shown that specific imaging modalities, for example USI, are more widely used in Europe compared with USA and Australasia [48, 49], which may be due to variations in the structure of musculoskeletal radiology training across countries [50]. This finding highlights the importance of ongoing international collaboration in this research field [51–54], with different researchers and institutions contributing different skill sets based on their specific knowledge of different imaging modalities.

The performance analysis confirms the continuing use of plain radiography to assess foot osteoarthritis, with studies using plain radiography having the highest citation rate per study, and seven of the top ten most cited studies employing this modality. Citations are regarded as a measure of the impact or influence of a publication within its field of research [20, 55, 56]. The IF of journals are also regarded as an indicator of the influence and quality of their published studies [30, 57]. In the current analysis, the median journal IF was 2.83 for included studies, which is a relatively high median for the field based on an analysis by SCI Journal in 2018 [58]. SCI Journal found that only 2% of journals have an IF of 10 or more, 13% with IF of 4 or more and 20% with an impact of 3 or more. An IF greater than 2.26 places a journal in the top 40% of medical journals, indicating that the published studies are influential in their field [30, 57]. However, the validity of the IF as an accurate, meaningful and useful measure of determining research quality is largely debated [59–63]. Despite these limitations, IF is the most commonly used and arguably the best existing metric for evaluating the bibliometric impact of published research [61].

The results of this study have several limitations. Firstly, a single database (Scopus®) was used for study identification and some peer-reviewed journals are not indexed in Scopus®. Although hand-searching of reference lists of included studies was undertaken to address this limitation, it is possible that not all studies employing musculoskeletal imaging to assess foot osteoarthritis were included. Secondly, the results generated from the search strategy may have been affected by not including individual foot joint names as keywords. Thirdly, non-English studies were excluded from the current analysis which may have resulted in an underrepresentation of the scientific productivity of non-English speaking countries in the current study. Fourthly, in order to provide a comprehensive analysis of all studies in this field, our analysis was not limited to studies in which imaging was used to assess a primary outcome measure. This therefore limits our ability to comment on studies specifically aiming to advance knowledge and progress research in this field. Finally, it should also be acknowledged that the majority of studies included in this analysis were representative of a lower level of evidence according to the OCEBM (Level 3 or 4 evidence) [32], and may not be considered as influential as studies of higher evidence.

In conclusion, published research employing imaging to assess foot osteoarthritis has increased substantially over the past four decades. Although plain radiography remains the gold standard and the most utilised modality for research of foot osteoarthritis, the emergence of MRI, CT and USI in the past two decades continues to advance research in this field. This study has also highlighted the importance of international collaboration in allowing researchers and institutions with different skill sets and knowledge to contribute to ongoing research utilising imaging to evaluate foot osteoarthritis.

## Supplementary Information

**Additional file 1.**

## Data Availability

All data used in this article can be found on the Scopus database using the search strategy outlined in the Methods section. Reasonable requests for data beyond that contained in the Additional Files will be considered by the corresponding author.

## Abbreviations

CT: Computed Tomography
MRI: Magnetic Resonance Imaging
USI: Ultrasound Imaging
EULAR: European League Against Rheumatism
IF: Impact Factor
OCEBM: Oxford Centre for Evidence-Based Medicine
