# Supplementary material for: A bibliometric analysis of published research employing musculoskeletal imaging modalities to evaluate foot osteoarthritis
Source: J Foot Ankle Res. 2022 May 20;15:39. doi: 10.1186/s13047-022-00549-0 (PMC9121542; doi:10.1186/s13047-022-00549-0)
Supplement: Supplementary file 1 — Additional file 1. [file 13047_2022_549_MOESM1_ESM.docx]

**Supplementary Files**

**Supplementary File 1** Search strategy

| **Scopus** | | |  |
| --- | --- | --- | --- |
| 1 | Subject term | Imaging | |
| 2 | Keywords | “diagnostic imaging” OR radiograph* OR x-ray* OR roentogram OR roentgenogram OR ultrasonograph* OR ultrasound OR sonograph* OR “power doppler” OR doppler OR tomograph* OR “x-ray computed” OR “computed tomograph*” OR ct OR mri OR “magnetic resonance imaging” OR “SPECT-CT” OR “single photon-emission computed tomography" | |
| 3 | Combine | 1 OR 2 | |
| 4 | Keywords | osteoarthrosis OR osteoarthriti* OR oa | |
| 5 | Keywords | foot OR feet OR podiatr* | |
| 6 | Combine | 3 AND 4 AND 5 | |

**Supplementary File 2.** PRISMA flow chart


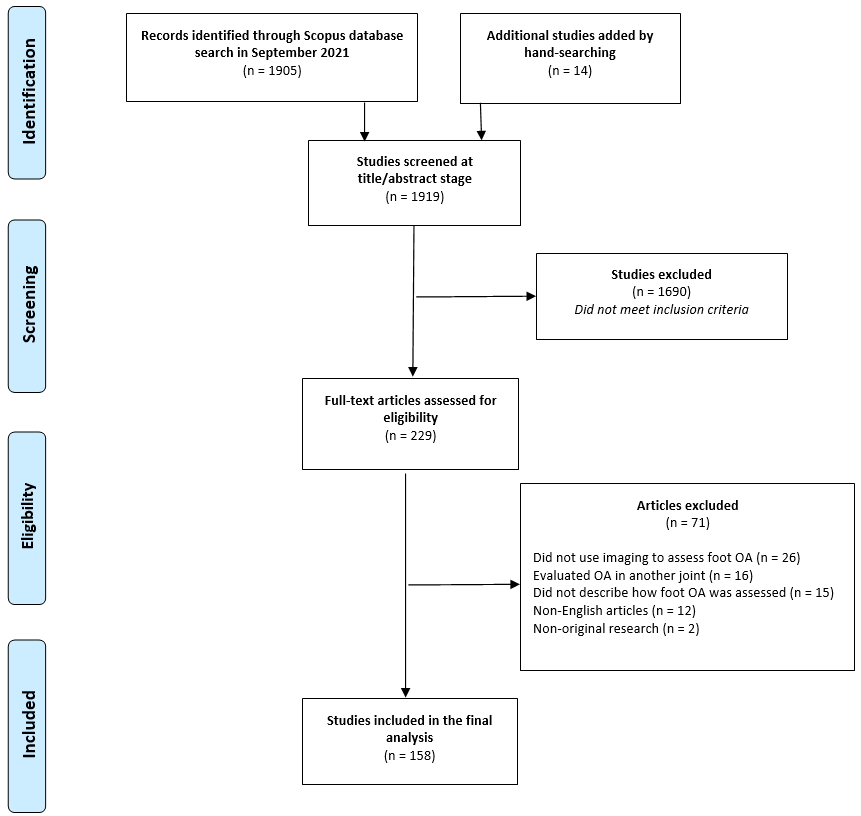


| **Supplementary File 3.** Foot joints examined across studies for each imaging modality^a^ | | | | | | |
| --- | --- | --- | --- | --- | --- | --- |
|  | |  | **Imaging modality** | | | |
| **Joint** | | **All studies** | **Plain radiography** | **CT** | **MRI** | **USI** |
| First metatarsophalangeal joint | | 64 | 57 | 5 | 5 | 11 |
| Lesser metatarsophalangeal joint (2-5) | | 9 | 7 | 1 | 1 | 1 |
| Subtalar joint | | 50 | 44 | 13 | 4 | 1 |
| Tarsometatarsal joint | | 24 | 21 | 5 | 1 | 1 |
| Naviculocuneiform joint | | 29 | 26 | 4 | 2 | 2 |
| Cuneometatarsal joint | | 18 | 18 | 0 | 0 | 1 |
| Talonavicular joint | | 45 | 42 | 8 | 3 | 2 |
| Calcaneocuboid joint | | 23 | 21 | 6 | 1 | 2 |
| Cuboid-navicular joint | | 3 | 3 | 0 | 0 | 1 |
| Cuboid-cuneiform joint | | 1 | 1 | 0 | 0 | 1 |
| Calcaneonavicular joint | | 2 | 2 | 0 | 0 | 0 |
| Interphalangeal joint | | 1 | 0 | 0 | 1 | 1 |
| Proximal Interphalangeal joint | | 3 | 2 | 1 | 0 | 1 |
| Distal Interphalangeal joint | | 3 | 2 | 1 | 0 | 1 |
| Foot joint not specified | | 6 | 5 | 0 | 3 | 2 |
|  | ^a^24 studies used >2 imaging modalities and 60 studies assessed >2 joints. | | | | | |

| **Supplementary File 4.** Top five most cited countries based on first author’s country across each imaging modality | | | | | | | | | | | | | | | |  |  |
| --- | --- | --- | --- | --- | --- | --- | --- | --- | --- | --- | --- | --- | --- | --- | --- | --- | --- |
|  | **Plain radiography** | | |  | **CT** | | |  | **MRI** | | |  | **USI** | | |  |  |
| Rank | Most cited country | Total citations | n | Average citations per study | Most cited country | Total citations | n | Average citations per study | Most cited country | Total citations | n | Average citations per study | Most cited country | Total citations | n | Average citations per study |  |
| 1. | USA | 1538 | 34 | 45.2 | USA | 216 | 4 | 54.0 | USA | 173 | 2 | 86.5 | Canada | 29 | 1 | 29.0 |  |
| 2. | Australia | 456 | 17 | 26.8 | Switzerland | 162 | 3 | 54.0 | Denmark | 49 | 1 | 49.0 | Italy | 20 | 2 | 10.0 |  |
| 3. | UK | 269 | 18 | 14.9 | Australia | 82 | 1 | 82.0 | UK | 34 | 3 | 11.3 | Germany | 8 | 1 | 8.0 |  |
| 4. | Switzerland | 203 | 7 | 29.0 | Germany | 42 | 4 | 10.5 | Germany | 18 | 2 | 9.0 |  |  |  |  |  |
| 5. | Germany | 82 | 7 | 11.7 | Japan | 23 | 1 | 23.0 | Italy | 11 | 1 | 11.0 |  |  |  |  |  |
| USA, United States of America | | | | | | | | | | | | | | | | | |

| **Supplementary File 5.** Characteristics of the ten most cited studies | | | | | |
| --- | --- | --- | --- | --- | --- |
| **Rank** | **Article** | **Journal** | **Imaging modality** | **Total citations** | **Annual citation rate/study** |
| 1 | Paley et al. 1993 (1) | Journal of Bone and Joint Surgery-Series A | Plain radiography | 202 | 6.97  11.00  5.93  4.42  9.73  4.00  2.36  6.77  5.80  3.04 |
| 2 | Dobbs et al. 2006 (2) | Journal of Bone and Joint Surgery-Series A | Plain radiography | 176 |  |
| 3 | Graves et al. 1993 (3) | Journal of Bone and Joint Surgery-Series A | Plain radiography | 172 |  |
| 4 | Mann et al. 1996 (4) | Journal of Bone and Joint Surgery-Series A | Plain radiography | 115 |  |
| 5 | El-Rashidy et al. 2011 (5) | Journal of Bone and Joint Surgery-Series A | MRI | 107 |  |
| 6 | Stephens et al. 1996 (6) | Foot and Ankle International | Plain radiography & CT | 104 |  |
| 7 | Martel et al. 1980 (7) | American Journal of Roentgenology | Plain radiography | 99 |  |
| 8 | Knupp et al. 2009 (8) | Journal of Orthopaedic Research | Plain radiography & CT | 88 |  |
| 9 | Menz et al. 2007 (9) | Osteoarthritis and Cartilage | Plain radiography | 87 |  |
| 10 | Frawley et al. 1995 (10) | Foot and Ankle International | Plain radiography & CT | 82 |  |

| **Supplementary File 6.** Characteristics of journals with >3 publications (n = 15 journals) | | | | | |
| --- | --- | --- | --- | --- | --- |
| **Journal** | **Studies, n (%)** | **Impact Factor** | **Total citations** | **Average citations per study** | **Average annual citation rate/study** |
| Foot and Ankle International | 24 (15.2) | 2.827 | 663 | 27.6 | 2.1 |
| Arthritis Care and Research | 11 (7.0) | 4.794 | 118 | 10.7 | 2.0 |
| Osteoarthritis and Cartilage | 10 (6.3) | 6.576 | 323 | 32.3 | 2.9 |
| Foot and Ankle Surgery | 9 (5.7) | 2.705 | 76 | 8.4 | 1.3 |
| Journal of Bone and Joint Surgery-Series A^a^ | 8 (5.1) | 5.284 | 931 | 116 | 6.0 |
| Journal of Foot and Ankle Surgery | 7 (4.4) | 1.286 | 82 | 11.7 | 1.7 |
| Journal of Bone and Joint Surgery-Series B^a^ | 5 (3.2) | NA | 127 | 25.4 | 1.6 |
| Injury | 4 (2.5) | NA | 31 | 7.8 | 1.0 |
| American Journal of Roentgenology | 3 (1.9) | 3.959 | 107 | 35.7 | 1.7 |
| Annals of the Rheumatic Diseases | 3 (1.9) | 19.103 | 161 | 53.7 | 5.9 |
| Clinical Rheumatology | 3 (1.9) | 2.98 | 28 | 9.3 | 1.6 |
| European Journal of Radiology | 3 (1.9) | 3.528 | 25 | 8.3 | 1.3 |
| Foot | 3 (1.9) | NA | 10 | 3.3 | 0.7 |
| Journal of Foot and Ankle Research | 3 (1.9) | 2.303 | 4 | 1.3 | 0.5 |
| Journal of Orthopaedic Science | 3 (1.9) | 1.601 | 25 | 8.3 | 1.5 |
| ^a^Series A = American volume, Series B = British volume; NA= 2020 IF not available | | | | | |

| **Supplementary File 7.** Top 10 most published authors and affiliations | | | | | | | |
| --- | --- | --- | --- | --- | --- | --- | --- |
|  | **Authors** | | | | **Affiliations^a^** | | |
| **Rank** | **Author** | **Any author appearances, n (%)** | **First author appearances, n (%)** | **Total citations** | **Affiliation** | **Studies, n (%)** | **Total citations** |
| 1 | Menz HB | 27 (17.1) | 10 (6.3) | 560 | La Trobe University | 27 (17.1) | 559 |
| 2 | Munteanu SE | 17 (10.8) | 4 (2.5) | 390 | Keele University | 19 (12.0) | 258 |
| 3 | Roddy E | 17 (10.8) | 1 (0.6) | 254 | University of Iowa | 6 (3.8) | 221 |
| 4 | Marshall M | 11 (7.0) | 0 (0) | 181 | University of Leeds | 5 (3.2) | 45 |
| 5 | Auhl M | 9 (5.7) | 0 (0) | 62 | New York University | 4 (2.5) | 155 |
| 6 | Landorf KB | 9 (5.7) | 0 (0) | 258 | University of Southampton | 4 (2.5) | 8 |
| 7 | Thomas MJ | 9 (5.7) | 2 (1.3) | 167 | Duke University | 3 (1.9) | 127 |
| 8 | Tan JM | 8 (5.1) | 0 (0) | 60 | Hospital or special surgery | 3 (1.9) | 89 |
| 9 | Zammit GV | 8 (5.1) | 2 (1.3) | 328 | University of Utah | 3 (1.9) | 63 |
| 10 | Rathod T | 6 (3.8) | 1 (0.6) | 171 | Oslo University Hospital | 3 (1.9) | 10 |
| ^a^Number of studies based on the affiliation of any listed author | | | | | | | |

**References**

1. Paley D, Hall H. Intra-articular fractures of the calcaneus. A critical analysis of results and prognostic factors. J Bone Joint Surg Am. 1993;75(3):342-54.

2. Dobbs MB, Nunley R, Schoenecker PL. Long-term follow-up of patients with clubfeet treated with extensive soft-tissue release. J Bone Joint Surg Am. 2006;88(5):986-96.

3. Graves SC, Mann RA, Graves KO. Triple arthrodesis in older adults. Results after long-term follow-up. J Bone Joint Surg Am. 1993;75(3):355-62.

4. Mann RA, Prieskorn D, Sobel M. Mid-Tarsal and Tarsometatarsal Arthrodesis for Primary Degenerative Osteoarthrosis or Osteoarthrosis after Trauma*. JBJS. 1996;78(9):1376-85.

5. El-Rashidy H, Villacis D, Omar I, Kelikian AS. Fresh osteochondral allograft for the treatment of cartilage defects of the talus: A retrospective review. J Bone Joint Surg Am. 2011;93(17):1634-40.

6. Stephens HM, Sanders R. Calcaneal malunions: Results of a prognostic computed tomography classification system. Foot Ankle Int. 1996;17(7):395-401.

7. Martel W, Stuck KJ, Dworin AM, Hylland RG. Erosive osteoarthritis and psoriatic arthritis: A radiologic comparison in the hand, wrist, and foot. AJR Am J Roentgenol. 1980;134(1):125-35.

8. Knupp M, Pagenstert GI, Barg A, Bolliger L, Easley ME, Hintermann B. SPECT-CT compared with conventional imaging modalities for the assessment of the varus and valgus malaligned hindfoot. Journal of Orthopaedic Research. 2009;27(11):1461-6.

9. Menz HB, Munteanu SE, Landorf KB, Zammit GV, Cicuttini FM. Radiographic classification of osteoarthritis in commonly affected joints of the foot. Osteoarthritis Cartilage. 2007;15(11):1333-8.

10. Frawley PA, Hart JAL, Young DA. Treatment outcome of major fractures of the talus. Foot Ankle Int. 1995;16(6):339-45.
